# Nephroblastoma-specific dysregulated gene *SNHG15* with prognostic significance: scRNA-Seq with bulk RNA-Seq data and experimental validation

**DOI:** 10.1007/s12672-024-00946-w

**Published:** 2024-03-25

**Authors:** Mengmeng Chang, Ding Li, Li Su, Chen Ding, Zhiyi Lu, Hongjie Gao, Fengyin Sun

**Affiliations:** 1 Department of Pediatric Surgery, Qilu Hospital of Shandong University, Jinan, China; 2 Department of Pediatrics, Qilu Hospital of Shandong University, Jinan, China

**Keywords:** ScRNA-Seq, Bulk RNA-Seq, Prognostic, Nephroblastoma, M2 macrophages

## Abstract

Wilms tumor (WT) is the most common malignancy of the genitourinary system in children. Currently, the Integration of single-cell RNA sequencing (scRNA-Seq) and Bulk RNA sequencing (RNA-Seq) analysis of heterogeneity between different cell types in pediatric WT tissues could more accurately find prognostic markers, but this is lacking. RNA-Seq and clinical data related to WT were downloaded from the Therapeutically Applicable Research to Generate Effective Treatments (TARGET) database. Small nucleolar RNA host gene 15 (*SNHG15*) was identified as a risk signature from the TARGET dataset by using weighted gene co-expression network analysis, differentially expressed analysis and univariate Cox analysis. After that, the functional mechanisms, immunological and molecular characterization of *SNHG15* were investigated at the scRNA-seq, pan-cancer, and RNA-seq levels using Gene ontology (GO), Kyoto Encyclopedia of Genes and Genomes (KEGG), ESTIMATE, and CIBERSORT. Based on scRNA-seq data, we identified 20 clusters in WT and annotated 10 cell types. Integration of single-cell and spatial data mapped ligand-receptor networks to specific cell types, revealing M2 macrophages as hubs for intercellular communication. In addition, in vitro cellular experiments showed that siRNAs interfering with *SNHG15* significantly inhibited the proliferation and migration of G401 cells and promoted the apoptosis of G401 cells compared with the control group. The effect of siRNAs interfering with *SNHG15* on EMT-related protein expression was verified by Western blotting assay. Thus, our findings will improve our current understanding of the pathogenesis of WT, and they are potentially valuable in providing novel prognosis markers for the treatment of WT.

**Supplementary Information:**

The online version contains supplementary material available at 10.1007/s12672-024-00946-w.

## Introduction

Nephroblastoma, also called Wilms tumor (WT), is an embryonic tumor prevalent in children under 5 years of age [1]. This disease accounts for 90% of kidney tumors in pediatric patients and 7% of all pediatric cancers. In recent years, this disease has an increasing incidence in China with each passing year, which has seriously threatened the life of children. Currently, this disease is clinically assessed mainly based on the pathological types of tumors. However, there are considerable limitations for assessing the prognosis of children with nephroblastoma [2]. Therefore, exploring the molecular mechanism of the occurrence and development of nephroblastoma is of great significance for improving the diagnostic efficiency and assessing the prognosis of children with nephroblastoma [3].

In recent years, high-throughput sequencing (HTS) technology has been widely used in various fields of biology and medicine [4]. Especially in the field of oncology, the rapid development of HTS technology and bioinformatics has "deciphered" the code of tumor cells and promoted targeted therapy and immunotherapy to achieve precision medicine [5]. ScRNA-seq allows researchers to investigate the variability and complex gene expression across all the individual cells instead of a more homogeneous expression profile from traditional bulk RNA sequencing of tissues. ScRNA-seq can reveal heterogeneity among different cells [6]. As a powerful tool for exploring tumor immune microenvironment (TIME), scRNA-seq plays a crucial role in revealing TIME maps, analyzing cell fate, and exploring cellular interactions [7].

LncRNAs are RNA molecules larger than 200 nucleotides, and it is differentially expressed in specific tissues and different types of tumors. Although lncRNAs can't encode proteins, they can perform epigenetic regulation, such as DNA methylation modification and histone modification. Besides, they can also affect DNA transcription and regulate gene expression through *cis*- and *trans*-actions, and hence they play a decisive role in the occurrence and development of tumors [8]. Further, lncRNAs closely correlate with cell proliferation and differentiation, and they can regulate life activities and the occurrence and development of various tumors. Thus, they can be considered effective biomarkers [9, 10].

The rapid development of gene chips, RNA sequencing, and other technologies has facilitated the widespread application of bioinformatic analysis techniques in tumor diagnosis and treatment. However, the identification of differentially expressed genes (DEGs) is highlighted in these methods, and the functional correlation between similarly expressed genes is ignored. Fortunately, this deficiency can be eliminated by WGCNA through constructing a co-expression network to explore the correlation between different gene modules and clinical manifestations [11]. In WGCNA, similarly expressed genes are clustered, their core genes are classified, and their correlation with sample characteristics (including clinicopathological indexes and treatment methods) is analyzed. With the assistance of WGCNA, the modules and genes related to the prognosis of diseases can be rapidly obtained from multiple transcriptomes, and the comparison of connectivity and genetic importance between modules can be performed [12]. Due to the fact that there is heterogeneity in nephroblastoma, the sensitivity and specificity of conventional differential expression in small samples are not high, and it is of little significance in clinical application. However, this problem can be eliminated by WGCNA.

In this study, scRNA-Seq and Bulk RNA-Seq data were integrated to analyze the heterogeneity between different cell types in pediatric Wilms' tumor (WT) tissue. WGCNA and other bioinformatics approaches were used to explore high-throughput sequencing data and clinical data from nephroblastoma patients in the TARGET database and to validate them in in vitro experiments. Finally, we analyzed the key gene *SNHG15* by in vitro experiments, revealing that it could serve as a new potential prognostic biomarker for WT.

## Materials and methods

### Data acquisition

The scRNA-seq data of GSE200256 was downloaded from the Gene Expression Omnibus (GEO) database(https://www.ncbi.nlm.nih.gov/gds) (Access date: September 11, 2022). Under the management of NCBI's Office of Cancer Genomics and Cancer Therapy Evaluation Program, the TARGET database is a special database for pediatric cancer registration. This database is publicly available, and the data records include clinical data and multiomics data of pediatric cancer patients. The RNA sequencing (RNA-seq) transcriptome data and clinical data of WT patients were retrieved from the TARGET database, containing a total of 129 WT and 6 healthy tissue samples. The uniformly normalized pan-cancer dataset was downloaded from the UCSC database (https://xenabrowser.net/) (Access date: September 15, 2022). The GSE66405 dataset in the GEO database can be employed to verify whether there are differences in the expression of *SNHG15* [13, 14].

### Identification of differentially expressed lncRNAs

The original expression data were pre-processed by the Limma function package in R software [15]. The human genome (hg38) and associated annotation file (version 31) were obtained from the Gencode database (https://www.gencodegenes.org) [16]. This annotation file was used to identify lncRNAs. The molecules with gene types being "lincrna", "antisense", "processed transcript", "sense_intronic", "TEC", "bidirectional promoter lncRNA", "sense_overlapping", "macrolncRNA" or "non coding" were defined as lncRNAs. When multiple probes corresponded to the same lncRNA, the average of their expression values. In this study, |Log2 (FC)|> 1 and corrected P < 0.05 were selected as the criteria for identifying DEGs [15].

### WGCNA

After abnormal outliers in TARGET data sets were eliminated, and the network construction and module clustering were performed. The WGCNA function package in R software was adopted to construct a scalefree gene coexpression network [11]. Pearson correlation matrix analysis was performed on all gene pairs to construct the weighted adjacency matrix. The adjacency matrix between genes were transformed into the topological matrix (TOM), which was a biologically relevant measure of gene similarity based on the co-expression correlation between two genes. Then, the correlation between a specific gene and all directly or indirectly associated genes was identified. The similarity between genes was calculated by the topological overlap method. These genes were classified by the dynamic tree cutting method, and modules were named by color. Subsequently, Pearson correlation analysis was performed to measure the correlation (P-value) of each module with histological stages, overall survival (OS), and relapse-free survival (RFS). The lncRNA (key lncRNA) in the gene module with the highest correlation coefficient was taken as a candidate prognostic molecular marker for the subsequent analysis.

### Prognostic role and validation of *SNHG15*

The candidate lncRNAs were subjected to survival analysis based on the clinical data sets in the TARGET database. WT samples were separated into low- and high-*SNHG15* subtypes, based on median *SNHG15* expression values in the dataset. The prognosis of patients with WT in the sub-group was scrutinized by Kaplan–Meier analysis, and the value of *SNHG15* expression for predicting the prognosis of patients with WT in the cohort was estimated using survival rate, ROC curves, and AUC values. Subsequently, Cox regression analyses were conducted to ascertain whether *SNHG15* expression was an independent prognostic biomarker in patients with WT [17]. Based on this, an independent dataset, GSE66405, downloaded from the GEO database, validated the differences in *SNHG15* expression in normal kidney tissues versus nephroblastoma. In addition, based on the starBase database (https://starbase.sysu.edu.cn/index.php) and LncACTdb3.0 data-base (http://bio-bigdata.hrbmu.edu.cn/LncACTdb/index.html), these candidate lncRNAs were analyzed in an attempt to further explore the potential mechanisms by which these lncRNAs affect clinical features [18, 19]. These candidate lncRNAs were subjected to GO functional annotation and KEGG signaling pathway enrichment analyses, and correlation analyses with key pathway molecules.

### Immunological characteristic analysis

The ESTIMATE algorithm was utilized to assess the immune cell infiltration and the stromal cell distribution, which contributes to clarifying the effect of the tumor microenvironment (TME) on tumor cells [20]. The CIBERSORT and ssGSEA [21] algorithms were utilized to assess the immune cell infiltration and response between the normal group and the WT group. The enrichment levels of 16 immune cells and 13 immune functions were further quantified with the ssGSEA algorithm to evaluate the immunological characteristics of both groups. Moreover, the potential immune checkpoints were also predicted in this study [22].

### Single-cell sequencing analysis

The original expression profile dataset (GSE200256) used for analysis was screened using the GEO public database. We first filtered the scRNA-seq data using the R package Seurat for data processing [23], setting each gene to be expressed in a minimum of 3 cells, and each cell to express at least 250 genes. The percentage of mitochondria and rRNA was calculated by the PercentageFeatureSet function and ensured that each cell expressed more than 500 genes and < 6000 genes, with < 10% mitochondrial content and at least 100 unique molecular identifiers (UMIs) per cell. Then, the data were normalized by log-normalization, and the FindVariableFeatures function was used to find highly variable genes. All the genes were scaled using the ScaleData function, and principal component analysis (PCA) downscaling was performed. Finally, the cells were clustered using the FindNeighbors and FindClusters functions to obtain cell subgroups. SingleR (v1.8.1), CellMarker database [24] and PanglaoDB database [25] were used for cell type annotation. In addition, functional enrichment of "HALLMARK" was performed on cancer cells with high/low *SNHG15* expression with the use of "irGSEA" and "GSVA" in R [26]. CellChat was used to explore the potential interactions between core cells. To explain the molecular mechanism of WT progression, pseudo-temporal analysis was performed using "monocle2", and CellChat was used to explore potential interactions between core cells [27].

### Analysis of *SNHG15* pan-cancer differentially expressed and RNA-modified genes

We downloaded the uniformly normalized pan-cancer dataset: TCGA TARGET GTEx (PANCAN, N = 19,131, G = 60,499) from the UCSC (https://xenabrowser.net/) database, from which we further extracted the expression data of *SNHG15* in each sample. Further, we extracted the expression data of *SNHG15* in each sample, and further we screened the samples from: Solid Tissue Normal, Primary Solid Tumor, Primary Tumor, Normal Tissue, Primary Blood Derived Cancer - Bone Mar-row, and Primary Blood Derived Cancer - Peripheral, we further performed log2(x + 0.001) transformation for each expression value, and finally we also excluded those with less than 3 samples in a single cancer species, and finally obtained the expression data of 34 cancer species. We further extracted the expression data of *SNHG15* and 44 class III RNA modification’s (m1A, m5C, m6A) genes in each sample, and next we calculated the Pearson correlation between *SNHG15* and five classes of immune pathways of marker genes [28].

### In vitro cell validation experiments

#### Cell line and cell transfection

G401 cells were purchased from Tianjin Haohe Biotechnology Co., Ltd. (Tianjin, China). G401 cells were maintained in McCoy' 5A medium with 10% foetal bovine serum (Gibco) and 1% penicillin–streptomycin at 37 ℃ in an incubator with 5% CO2. One day before transfection, cells were grown in 6-well plates at 2.5 × 105/well and cultured overnight. When the cells were cultured to 50–60% fusion, the recombinant plasmid was transfected into the cells according to the instructions of LipofectamineTM8000. Subsequent operations were performed 48 h after transfection. The small interfering RNA (siRNA) was purchased from General Biologicals (Anhui, China), and the target sequence: F: 5′-CCUUGAGUCUCAUGUUUCAA-3′, R: 5′-UUGAACAUGAGACUCAAGG-3′.

#### Real-time quantitative PCR (RT-qPCR)

The total RNA was extracted by the TRIzol reagent (Invitrogen). The concentration and purity of RNAs were estimated by reading the absorbance at 260 nm and 280 nm. SYBR Green Real-Time PCR Master Mix (Takara, Japan) and Chromo 4 Real-Time PCR Detector (Bio-Rad, USA) were used to perform RT-qPCR. The primer sequences used in this study are listed in Supplementary table 1.

#### Cell viability assay

Calculate the amount of diluent needed to be added according to the density of 5000 cells/ml, add the appropriate amount of complete medium and mix it well by pipetting. The mixed cells were inoculated into 96-well plates at 200 μl/well and placed in an incubator at 37 ℃ with 5% CO2 overnight. CCK-8 solution (#CA1210, Solarbio, China) (10 μl/well) was added. After incubation for 2 h at 37 °C in an incubator with 5% CO2, the absorbance (OD) was measured at 450 nm by Tecan Spark TM10M (Tecan, Switzerland). The experiment was repeated 3 times and the average value was taken.

#### Cell scratch assay and transwell assay

Cell migration was evaluated by the scratch assay and the transwell test. Cells with a confluent density of 95–100% in a 6-well tissue culture plate were manually scraped with a sterile 10 μl plastic pipette head. These scratches were imaged within 48 h to measure wound closure and cell movement to the scratches. In the transwell test, an 8 μM transwell chamber (Millipore) was placed on a 24-well plate. Specifically, 100 μl of serum-free medium was added to the upper chamber (1 × 10^4^ cells/well), and 600 μl of medium containing 20% FBS was added to the lower chamber. After the serum-free culture for 36 h, these cells were fixed with methanol and stained with 0.1% crystal violet. Finally, the number of cells in the transwell assay was counted with ImageJ to measure the migration of cells in the cell scratch assay.

#### Western blotting

After 48 h of transfection, the cells of each group were collected, and the total protein was extracted and quantified. After that, the same amount of protein sample was subjected to sodium dodecyl sulfate–polyacrylamide gel electrophoresis (SDS-PAGE). The isolated proteins were transferred to polyvinylidene fluoride (PVDF) membranes (Bio-Rad, Hercules, CA, USA). After the film was sealed with 5% skim milk at room temperature for 1 h, the sample was incubated overnight with primary antibody (1:5000) at 4 ℃, and then incubated with secondary antibody (1:3000) at room temperature for 2 h. The Enhanced Chemiluminescence (ECL) detection kit (Millipore, USA) was used for relevant detection. TBST was washed three times, each time for 10 min. super-enhanced chemiluminescence (ECL) detection kit (Millipore, USA) was used to develop the images. The images were captured by a Bio-Rad gel imaging system and repeated three times, and the results were analyzed by ImageJ software.

#### Flow cytometry

Carefully collect the cell culture into a centrifuge tube and set aside, digest the cells, add the previously collected cell culture, blow down all the adherent cells and gently blow out the cells. Collect again into a centrifuge tube. After centrifugation, discard the supernatant, collect the cells, wash twice with PBS, follow the kit instructions, detect the cell cycle using the Cell Cycle and Apoptosis Assay Kit (#C1052, beyotime) and the CytoFLEX S Flow Cytometry System (USA), the experiments were performed in triplicate, repeated twice, and the data were processed using FlowJo software.

### Statistical methods

The survival analysis was conducted with the assistance of the survival, survminer, and PROC packages in R software, and the visualization was realized by the ggplot2 and forestplot packages in R software. The log-rank test was carried out, and the Kaplan–Meier survival analysis was performed to compare the survival difference between both groups. The time-dependent ROC analysis was performed to compare the accuracy of lncRNA prediction results. In terms of the Kaplan–Meier curve, the P-value and hazard ratio (HR) with a 95% confidence interval (CI) were obtained by the log-rank test and univariate Cox regression. The significant difference between both groups was determined by the Shapiro–Wilk normality test, independent samples T test, and Wilcoxon test. P < 0.05 indicated that the difference was statistically significant. All the above analysis methods were performed through R software (v4.2.1).

## Results

### Identification of DEGs

**Fig. 1:**
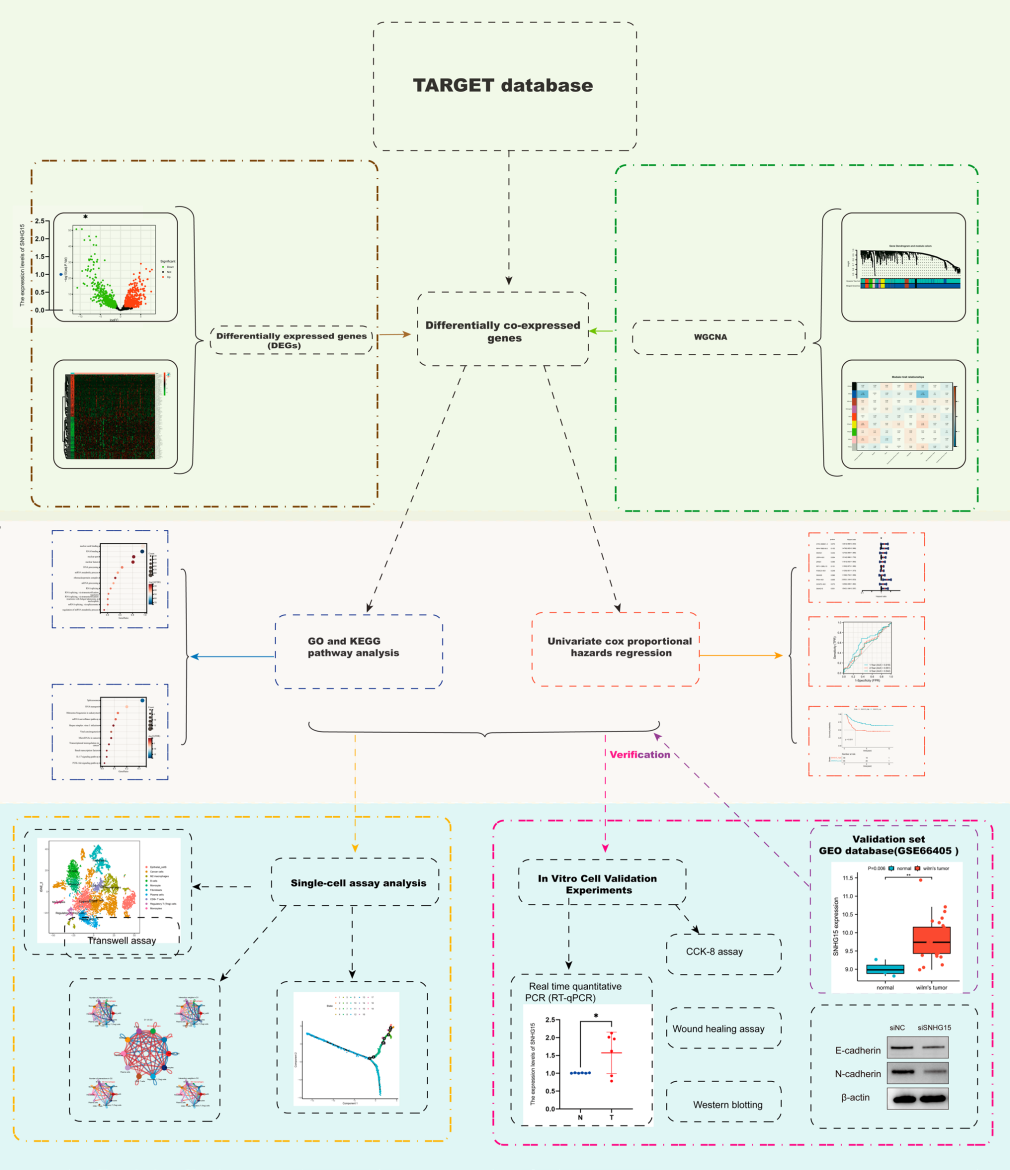
The flowchart of this study

**Fig. 2:**
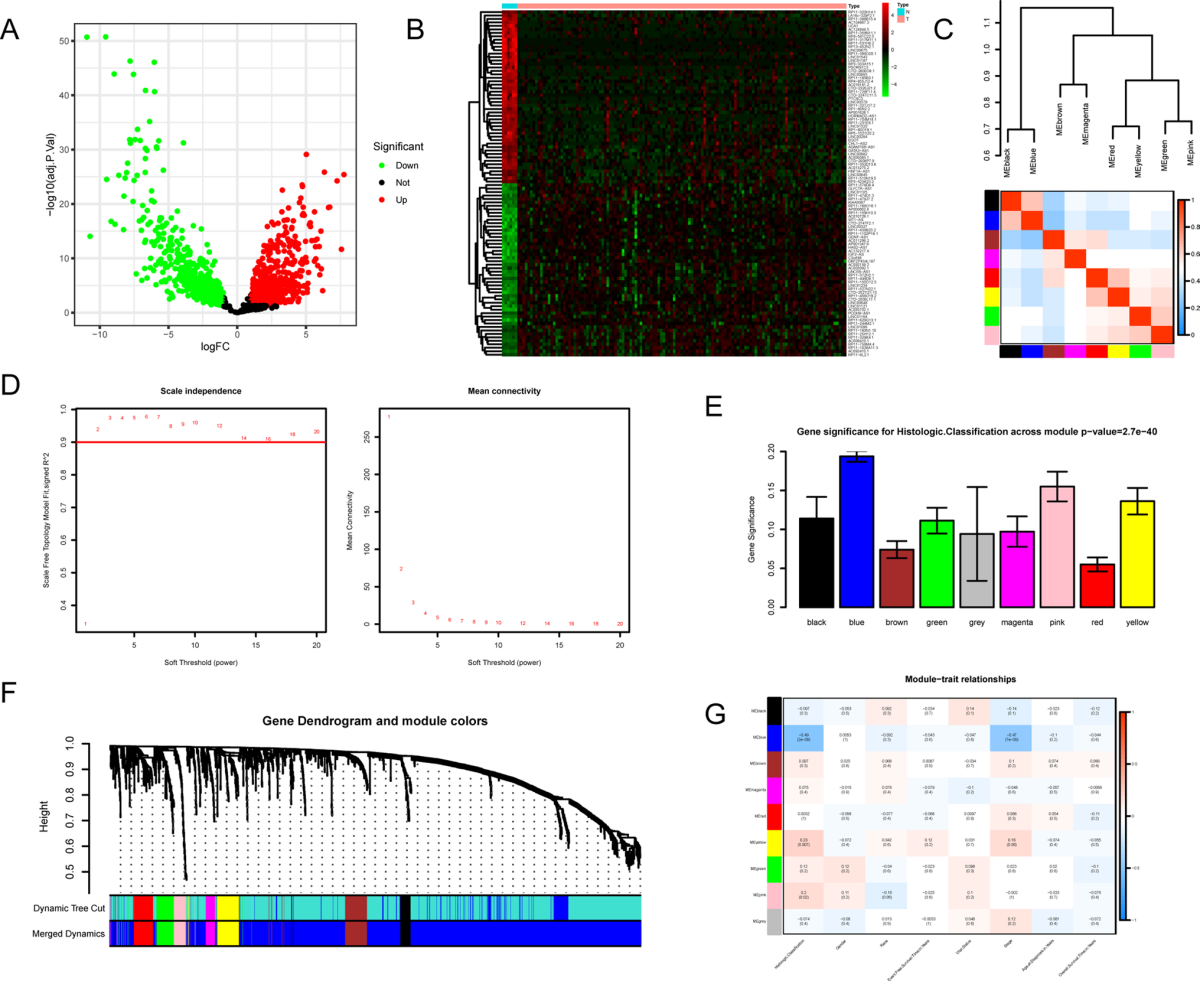
Identification of differential genes and WT-related genes were screened by WGCNA. **A** Volcanic map of DEGs; **B** heat map of DEGs; **C** heat map analysis of correlation between modules for β = 4; **D** analysis of the scale-free index for various soft-threshold powers (β); **E** Connectivity distribution histogram at β = 4; **F** Cluster dendrogram of the co-expression network modules (1-TOM); **G** Heat map of the correlation between characteristic gene modules and different clinical data of nephroblastoma

The workflow pertaining to the present study was shown in Fig. 1. The data sets related to nephroblastoma were downloaded from the TARGET database, and a total of 1839 DEGs were identified, including 1087 up-regulated genes and 752 down-regulated genes. Subsequently, the volcanic map and heat map of these DEGs were plotted (Fig. 2A, B). The expression data profiles of DEGs and their clinical data would be used for the subsequent WGCNA.

### Construction of the gene co-expression network

The gene co-expression network was constructed with the assistance of the WGCNA package in R software. It was found that outliers were not required to be eliminated, and hence all 1904 DEGs were included in WGCNA. Given the construction of the scale-free network and the moderate retention of average connectivity, β = 4 was selected to construct the co-expression network (the correlation coefficient = 0.98 was selected as the standard), as shown in Fig. 2C and D. All DEGs were divided into 9 modules based on WGCNA (Fig. 2E, F). In order to infer the clinical relevance of these genes, these gene modules were combined with the clinical data of patients with nephroblastoma. Tumor staging and survival data of patients were important evaluation indexes for selecting functional modules. The results demonstrated that the eigenvalue of the yellow module highly correlated with the histological type and pathological stage of patients with nephroblastoma, as shown in Fig. 2G. In order to explore the molecular markers related to prognosis, these lncRNAs in this module were selected for the subsequent survival analysis.

### Identification of LncRNAs related to the overall survival of patients with nephroblastoma

**Fig. 3:**
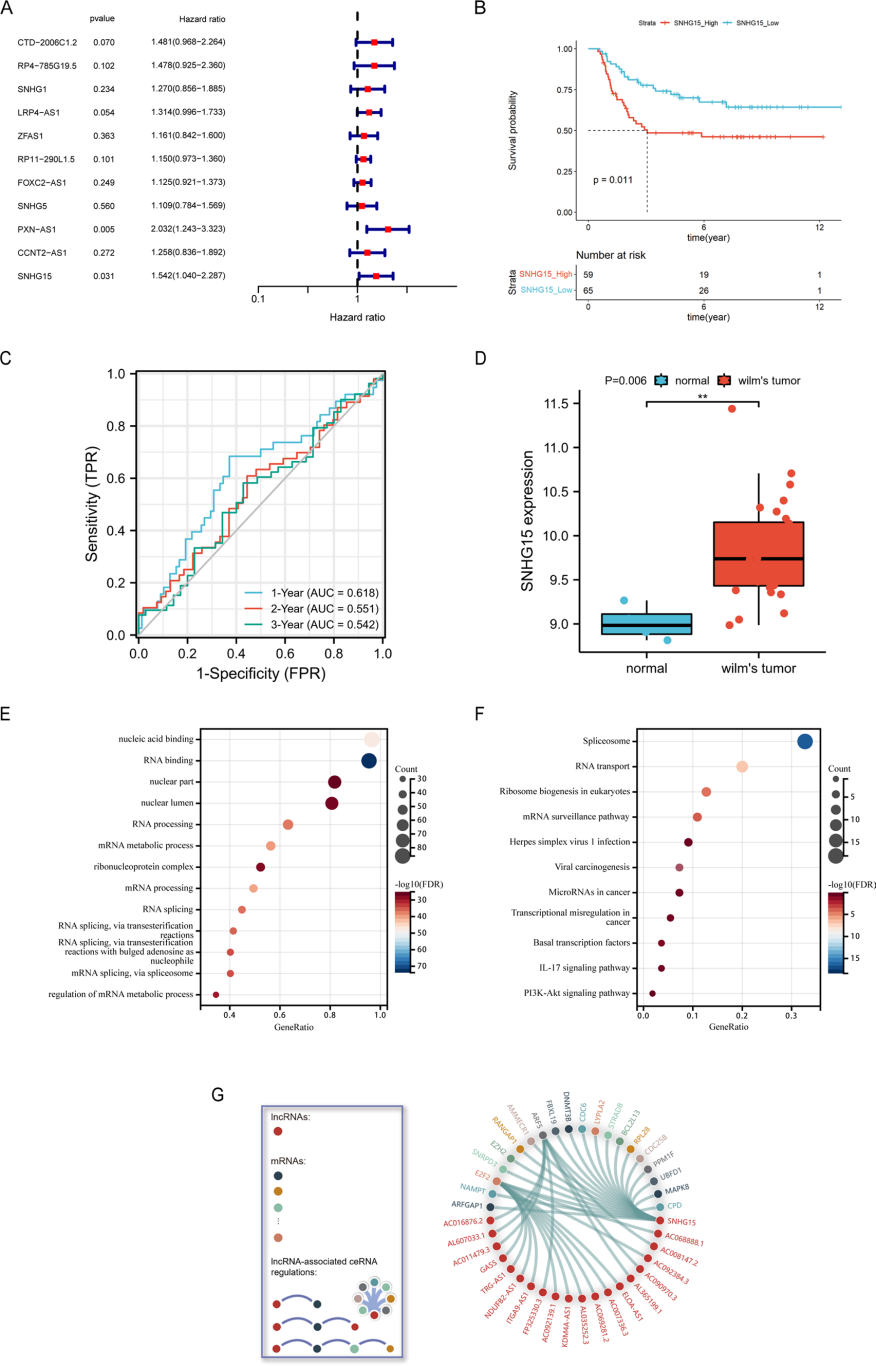
Clinical characterization and functional enrichment analysis of *SNHG15*. **A** Univariate Cox regression analysis of Overall survival and forest plots; **B** Kaplan–Meier curve result. **C** The AUC of the prediction of 1, 2, and 3-year survival rates of WT; **D** Expression of *SNHG15* in the validation set (GSE66405); GO function enrichment analysis bubble chart (**E**) and KEGG signaling pathway enrichment analysis bubble chart (**F**) of *SNHG15*-related mRNAs in the starBase data-base; **G** The LncACTdb3.0 database was adopted to analyze *SNHG15*-related ceRNAs and plot the ceRNA correlation circle diagram

The modules with a strong correlation with the prognosis of patients with nephroblastoma were selected. Then, the module significance (MS) of each module was calculated after each module was correlated with clinical features. The higher the MS value, the more important the module. Based on MS comparison, the modules with a strong correlation with a certain clinical feature were regarded as hub modules. Subsequently, the gene significance (GS) and module membership (MM) were calculated. In the hub modules, the genes with │MM│ > 0.8 and │GS│ > 0.2 were regarded as candidate hub genes. As a result, 11 candidate hub genes were obtained, including CTD-2006C1.2, RP4-785G19.5, SNHG1, LRP4-AS1, ZFAS1, RP11-290L1.5, FOXC2-AS1, SNHG5, PXN-AS1, CCNT2-AS1, and *SNHG15*. The clinical data of patients with nephroblastoma were downloaded from the TARGET database. The Kaplan–Meier survival analysis was performed on the lncRNAs of 11 candidate hub genes with the assistance of the survival package in R software. These patients with nephroblastoma were divided into two groups according to the best cut-off value of each lncRNA expression value. The Kaplan–Meier survival analysis results revealed that the overall survival rate of patients in the *SNHG15* high expression group was lower (P = 0.011), as shown in Fig. 3B. The time-dependent ROC curve indicated that *SNHG15* was accurate in predicting the 1-year, 2-year, and 3-year survival rates, and the area under the curve (AUC) was 0.618, 0.551, and 0.542, respectively (Fig. 3C). The univariate Cox regression analysis results suggested that *SNHG15* (HR = 1.196, P = 0.008) can be regarded as an independent prognostic factor (Fig. 3A).

### Expression and function prediction of *SNHG15* in nephroblastoma

The expression of *SNHG15* in nephroblastoma was verified with another independent data set (GSE66405) from the GEO database (https://www.ncbi.nlm.nih.gov/). The statistical analysis results suggested that the average expression level in the normal group and the WT group was 9.012 ± 0.197 and 9.844 ± 0.555, respectively. The independent samples T test results suggested that the average expression level in the WT group was higher than that in the normal group (Fig. 3D). The difference between both groups was 0.831 (0.253–1.41). The expression level of *SNHG15* in nephroblastoma was higher than that in normal kidney tissues, and there was a significant difference (t = 2.934, P = 0.006). The *SNHG15*-related mRNAs were predicted based on the StarBase database and LncACTdb3.0 database. Besides, the functional enrichment analysis and signal pathway enrichment analysis were carried out on the downstream target genes to infer the function of *SNHG15*, and the results were visualized (Fig. 3G). According to GO analysis, the down-stream target genes of *SNHG15* were mainly involved in methyltransferase activity, DNA modifying enzyme, post-transcriptional regulation of gene expression, regulation of cellular amide metabolism, mRNA synthesis, and the processing, splicing and binding of RNA (Fig. 3E). According to KEGG analysis, the downstream target genes were mainly involved in splice formation, *PI3K/AKT* signaling pathway [29], *IL-17* signaling pathway, *AMPK* signaling pathway, and cell cycle and apoptosis regulation, as shown in Fig. 3F. The *PI3K/AKT* signaling pathway is an intracellular signal transduction pathway, and it can respond to extracellular signals and promote metabolism, proliferation, cell survival, growth, and angiogenesis.

### Immune infiltration characteristics of patients with high and low levels of *SNHG15*

**Fig. 4:**
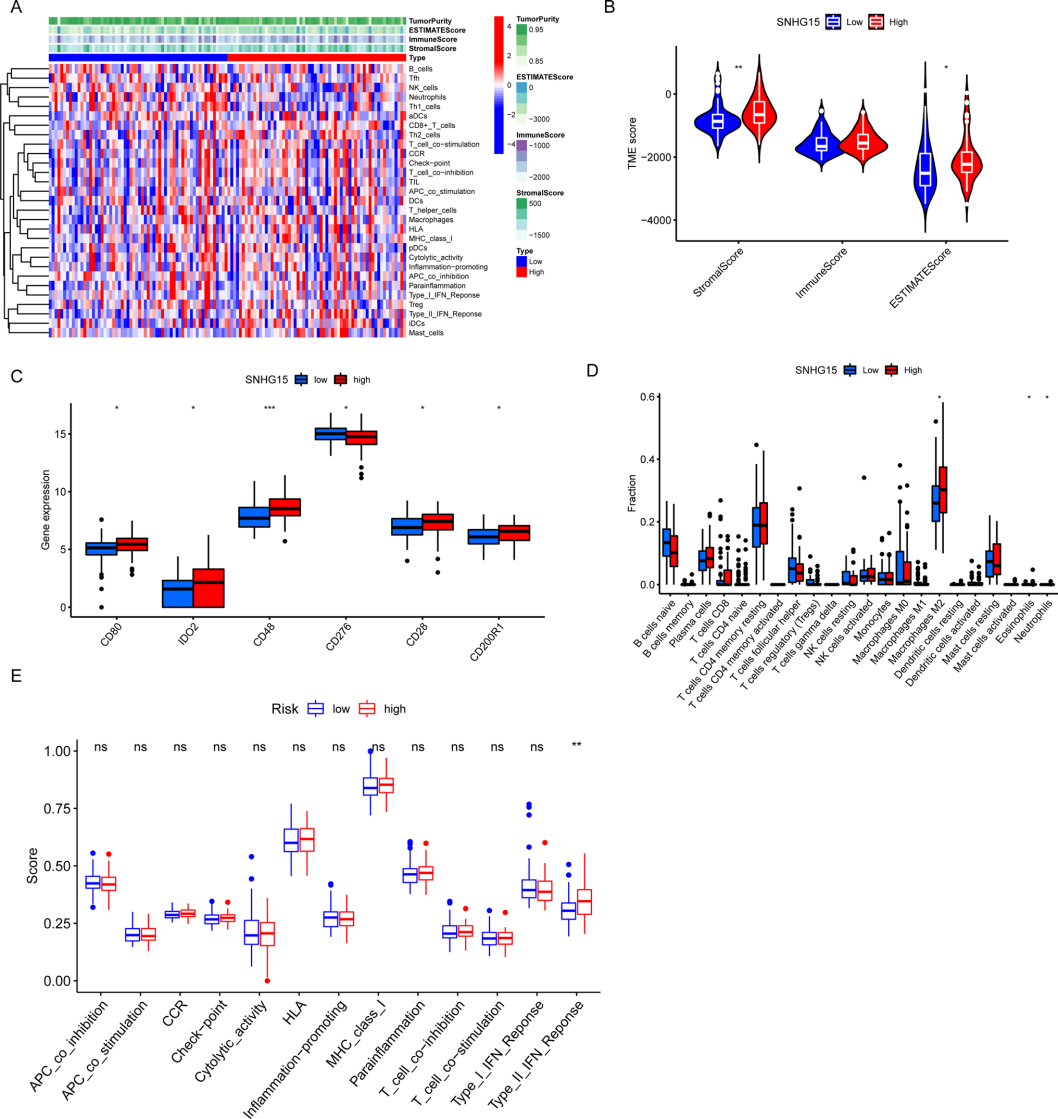
Analysis of the tumor immune microenvironment in high- and low- expression *SNHG15* groups. **A** Heat map of the immune status of patients in both groups based on the ssGSEA algorithm; **B** boxplot of immune scores between both groups; **C** boxplot of immune checkpoint expression between both groups; **D** boxplot of immune cell infiltration level between both groups; **E** boxplot of differences in immune function between both groups (*P < 0.05; **P < 0.01; ***P < 0.001)

To reveal differences of immune infiltration in patients with high and low levels of *SNHG15*, we evaluated the proportions of stromal and immune cells of patients in the TARGET cohort, and found that stromal and tumor purity were higher in patients with high *SNHG15* expression (Fig. 4A, B). Then, the relationship between *SNHG15* and the immune component of patients in the WT group was assessed by the ESTIMATE and ssGSEA algorithms. The results indicated that there was a significant difference in the immune cells and immune function between both groups. B cells, M0 and M1 macrophages were found to be fewer in number while M2 macrophages, eosinophils, and neutrophils were more abundant in the patients with high *SNHG15* expression. Moreover, the immune checkpoints were identified and there were significant differences in *CD80, CD48, IDO2, CD276, CD28*, and *CD200R1* between both groups (Fig. 4C–E) [30].

### Identification of WT cell subtypes

**Fig. 5:**
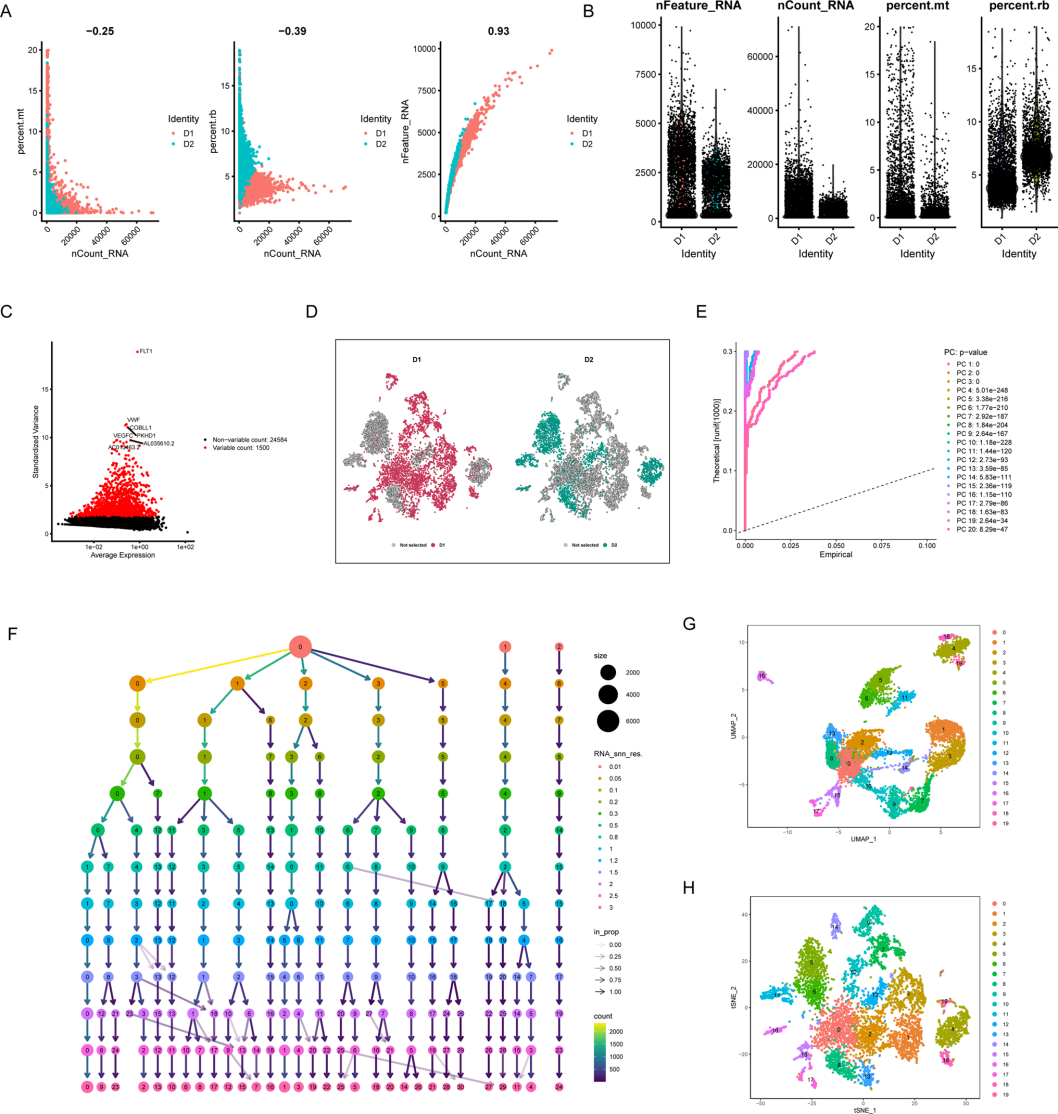
The scRNA-seq data (GSE200256) used for analysis was filtered. **A**, **B** The scRNA-seq data were filtered to filter out ineligible cells for subsequent analysis; **C** the variance plot shows the variation of gene expression in all cells of WT. The red dots represent highly variable genes and the black dots represent non-variable genes; **D** PCA showed a clear separation of cells in WT; **E**, **F** PCA identified the top 20 PCs at P < 0.05; the core cells were classified into 19 independent cell clusters using the Uniform Manifold Approximation and Projection (UMAP) (**G**) and t-distributed Stochastic Neighbor Embedding (t-SNE) algorithm (**H**)

The scRNA-seq data (GSE200256) used for analysis was filtered using the GEO database. We first filtered the scRNA-seq data using the R package "Seurat" for data processing, which filtered out unqualified cells for subsequent analysis (Fig. 5A, B). Then, the FindVariableFeatures function was used to find highly variable genes, and we found that 1500 genes were highly variable (Fig. 5C). The data were normalized by log-normalization. All genes were scaled using the ScaleData function and subjected to principal component analysis (PCA) downscaling, single-cell samples were scattered and distributed with logical results (Fig. 5D). Meanwhile, in PCA, we also selected 20 principal components (PCs) [31] with P.value less than 0.05 for subsequent analysis (Fig. 5E, F). Then, the core cells were classified into 19 independent cell clusters using the Uniform Manifold Approximation and Projection (UMAP) and t-distributed Stochastic Neighbor Embedding (t-SNE) algorithm (Fig. 5G, H) [32].

**Fig. 6:**
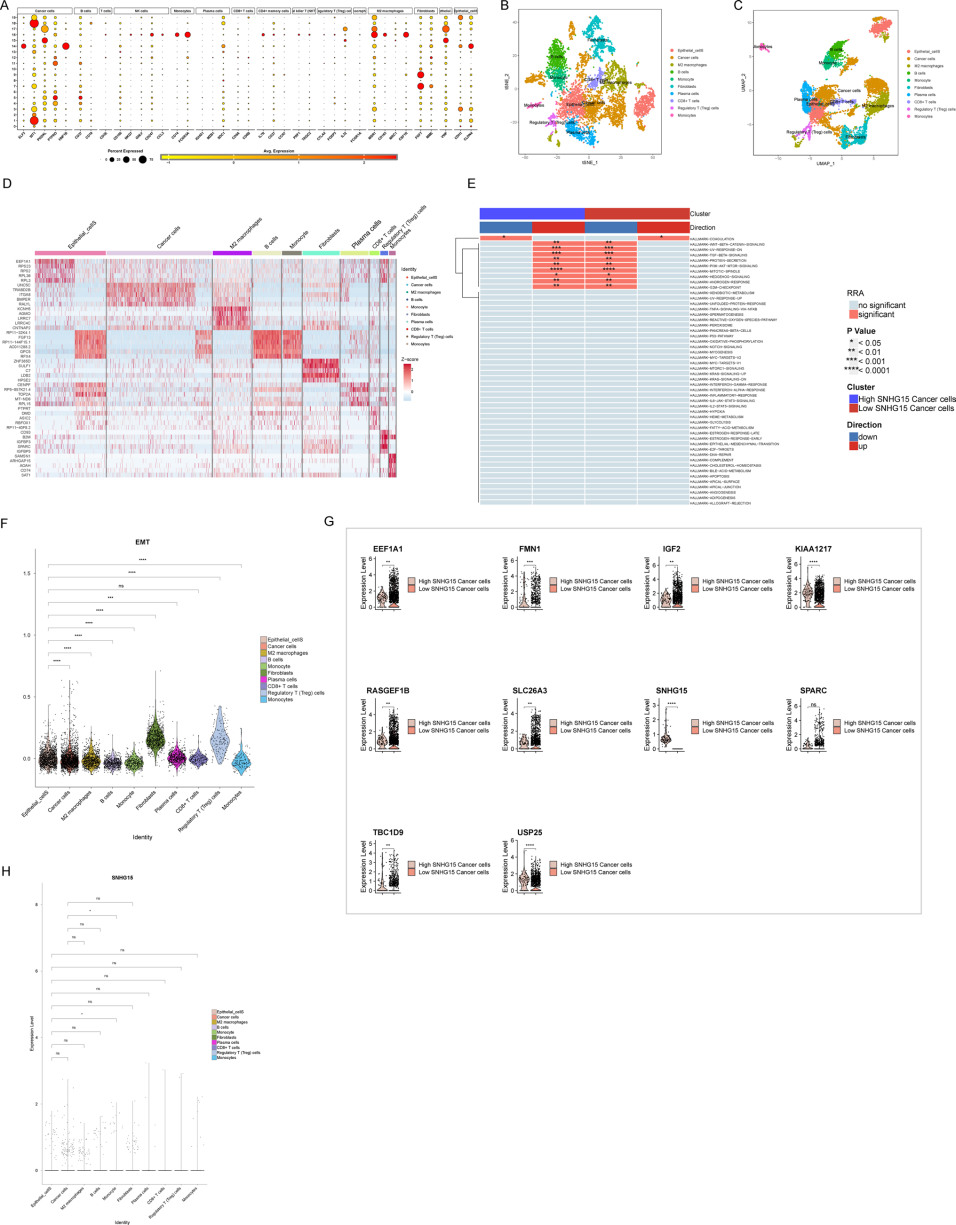
Annotation of the different cell clusters yielded 10 cell clusters. **A** Bubble plots of expression levels of marker genes for each cell cluster; **B**, **C** ten cell clusters were annotated based on the expression of marker genes; **D** Heatmap of specific gene markers in each cell type; **E** functional enrichment of "HALLMARK" was per-formed on cancer cells with high/low *SNHG15* expression. **F** EMT among cell types exhibits het-erogeneity of violin plots; G. Differentially expressed genes between tumor cells with high/low *SNHG15* expression; **H** *SNHG15* exhibited heterogeneity among different cell types, with the largest proportion of tumor cells and M2 macrophages

The different cell clusters were annotated by finding marker genes through the "singleR" package (v1.8.1), CellMarker database, and PanglaoDB database (Fig. 6A), resulting in 10 cell clusters, namely B cells, cancer cells, M2 macrophages, regulatory T cells, plasma cells, monocytes, CD8+ T cells, monocytes, fibroblasts, and epithelial cells (Fig. 6B, C) [33]. Specific gene marker expression in each cell type was shown in the form of a Heatmap (Fig. 6D).

In addition, functional enrichment of "HALLMARK" was performed on cancer cells with high/low *SNHG15* expression with the use of "irGSEA" and "GSVA" in R. GSVA analysis implied that “*wnt/β*-catenin signaling”, “*PI3K‐AKT‐MTOR* signaling”, and “*TGF-β* signaling” were enriched in the cells with high *SNHG15* expression (Fig. 6E). These results suggested that the high expression of *SNHG15* in cancer cells might play a role in promoting M2 macrophage infiltration through these pathways, thus affecting prognosis.

Epithelial-mesenchymal transition (EMT) occurs normally throughout development, and dysregulation of EMT can lead to tumorigenesis [34]. EMT was shown to exhibit heterogeneity among different cell types (Fig. 6F). In this study, we found that *SNHG15* exhibited heterogeneity among different cell types (Fig. 6G), with the largest proportion of tumor cells and M2 macrophages. The difference in the expression levels of tumor cells between the *SNHG15* high expression group and the *SNHG15* low expression group was statistically significant, and differentially expressed genes were visualized in the two groups (Fig. 6H).

### Intercellular interactions in nephroblastoma

**Fig. 7:**
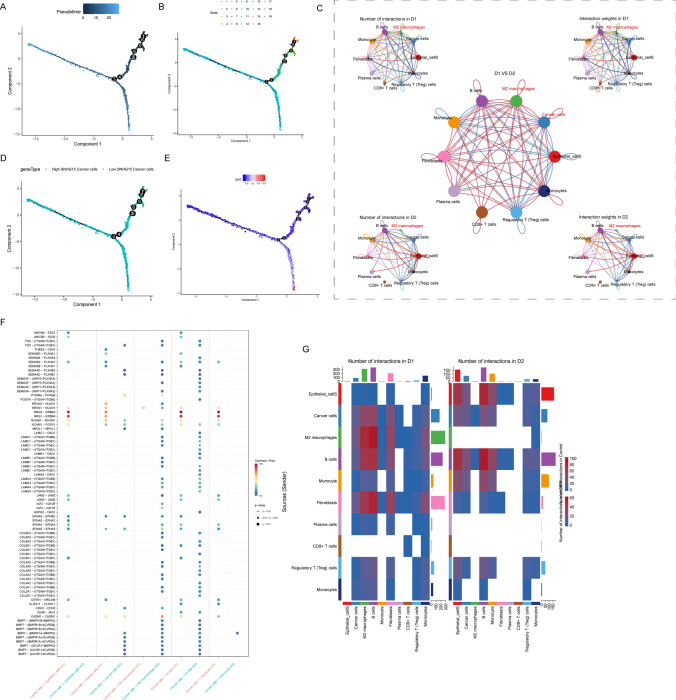
Intercellular interactions in nephroblastoma. **A**, **B** Trajectory analysis of three WT cell subpopulations with different differentiation patterns; **C** number and strength of interactions be-tween key cells; **D**, **E** epithelial cells differentiate earlier into two branches, one of which is dominated by EMT, and the other branch gradually differentiates in multiple directions and is dominated by high *SNHG15*-expressing tumor cells and M2 macrophages; Bubble charts (**F**) and heat maps (**G**) visualizes the number of potential ligand-receptor pairs in key cells

Pseudo-temporal analysis was performed separately for all clusters annotated in order to explore their differentiation directions with the Monocle 2 algorithm. The results showed that WT cells gradually followed 3 directions of differentiation (Fig. 7A, B). Epithelial cells divided earlier than other cells and differentiated into two branches, one of which was dominated by EMT (Fig. 7E), and the other branch gradually differentiated in multiple directions, dominated by tumor cells highly ex-pressing *SNHG15* and M2 macrophages (Fig. 7D). Furthermore, we inferred intercellular communication networks to predict intercellular communication according to specific pathways and ligand receptors. The Heatmap of the number of ligand-receptor pairs showed that cellular communication occurred more frequently in M2 macrophages, B cells, tumor cells, and epithelial cells (Fig. 7C, G).

Specifically, the frequency and intensity of interactions between cancer cells and M2 macrophages, and between cancer cells and epithelial cells were high (Fig. 7G). In addition, plasma cells, CD8+ T cells had relatively few interactions with other cells. The study of ligand-receptor pairs showed that the key receptor-ligand pairs between macrophages and tumor cells were mainly *NRXN3-NLGN1/NRXN1-NLGN1/NRG3-ERBB4/NRG1-ERBB4* (Fig. 7F).

### Analysis of *SNHG15* pan-cancer differentially expressed and RNA-modified genes

**Fig. 8:**
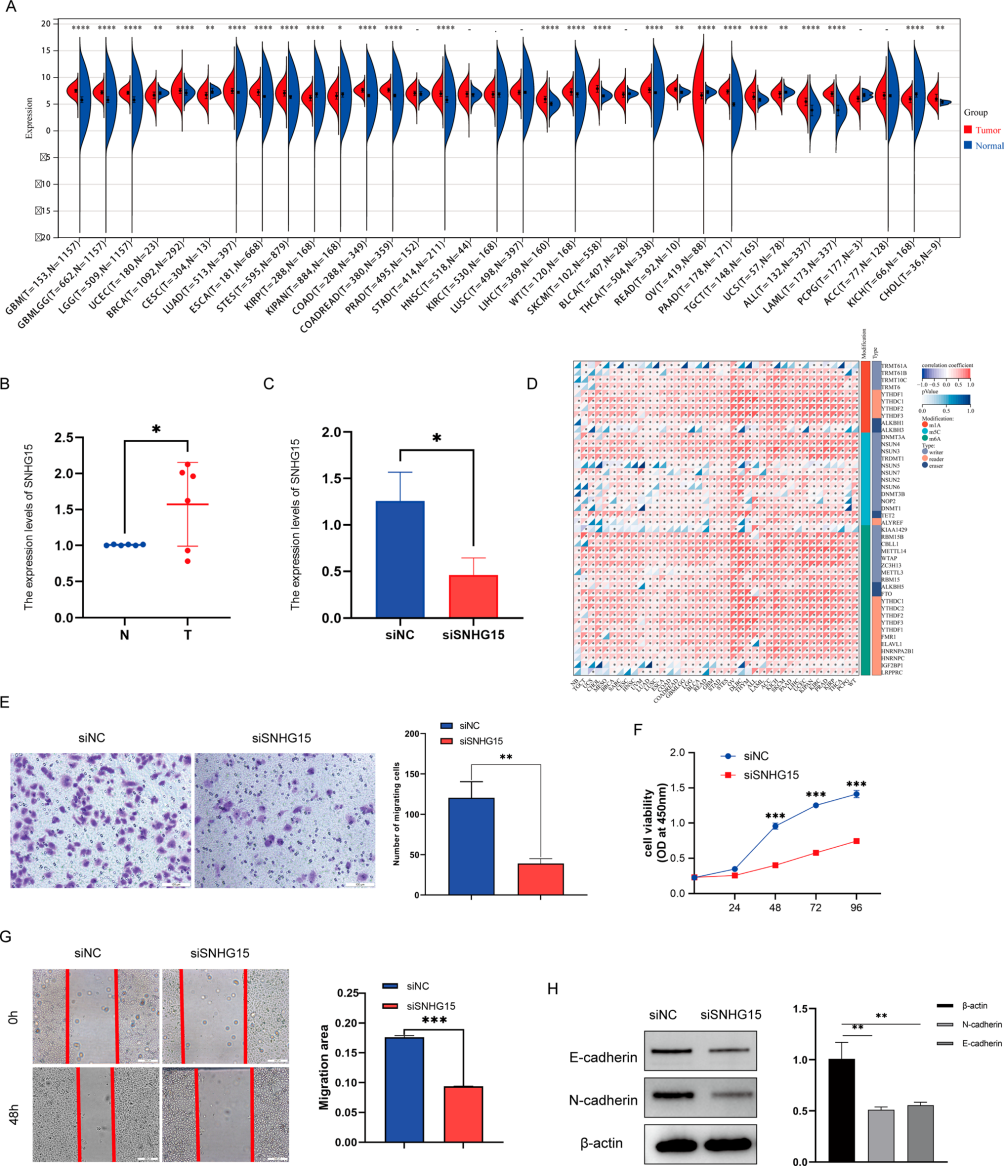
Validation of *SNHG15* from a pan-cancer and experimental perspective. **A** Analysis of *SNHG15* expression in pan-cancer; **B** the expression of *SNHG15* was measured by qRT-PCR in WT tissues and paired normal tissues; **C** the siRNA interference of *SNHG15* significantly inhibited the expression of *SNHG15* compared with the control group; **D** *SNHG15* is closely linked to marker genes for three classes of RNA modifications (m1A, m5C, and m6A) genes; **E** transwell assay, si*SNHG15* reduced the invasion and migration of nephroblastoma cells; **F** study of the effect of *SNHG15* on the proliferation and viability of nephroblastoma cells using CCK-8 assay; **G** wound healing assay: siRNA interference with *SNHG15* expression significantly inhibits the proliferation and migration of G401 cells; **H** the expression of EMT-related core proteins was examined by western blotting

We calculated the expression differences between normal and tumor samples in each tumor using R software and performed differential significance analysis using unpaired Wilcoxon Rank Sum and Signed Rank Tests (Fig. 8A). We observed significant upregulation in 20 tumors such as GBM (Tumor:7.39 ± 0.53, Normal:5.73 ± 1.46, p = 8.0e−63), GBMLGG (Tumor: 7.12 ± 0.52, Normal: 5.73 ± 1.46, p = 3.8e−156), LGG (Tumor:7.04 ± 0.49, Normal:5.73 ± 1.46, p = 5.1e−123), BRCA (Tumor. 7.36 ± 0.81, Normal: 7.14 ± 0.72, p = 5.9e−9), LUAD (Tumor: 7.40 ± 0.78, Normal: 7.09 ± 0.93, p = 9.9e−12), ESCA (Tumor: 7.11 ± 0.80, Normal: 6.24 ± 1.32, p = 2.0e−38), STES (Tumor: 6.97 ± 0.86, Normal: 6.09 ± 1.44, p = 1.9e−78), COAD (Tumor: 7.50 ± 0.55, Normal:6.34 ± 1.80, p = 2.8e−75), COADREAD (Tumor: 7.53 ± 0.56. Normal: 6.37 ± 1.78, p = 1.6e−87), STAD (Tumor: 6.90 ± 0.87, Normal: 5.59 ± 1.68, p = 9.8e−48), LIHC (Tumor:5.80 ± 0.90, Normal: 4.99 ± 0.56, p = 2.1e−27), WT (Tumor: 7.16 ± 0.86, Normal: 6.43 ± 1.55, p = 5.9e−9), SKCM (Tumor:7.69 ± 1.16, Normal: 6.49 ± 0.38, p = 1.5e−31), THCA (Tumor:7.49 ± 0.78, Normal:6.99 ± 1.00. p = 6.9e−33), READ (Tumor: 7.64 ± 0.58, Normal: 7.10 ± 0.47, p = 2.0e−3), PAAD (Tumor:7.19 ± 0.68, Normal: 4.76 ± 1.71, p = 1.9e−53), TGCT (Tumor: 6.35 ± 1.10. Normal: 5.75 ± 0.47, p = 3.0e−13), ALL (Tumor: 5.34 ± 1.10, Normal: 3.66 ± 1.34, p = 1.3e−29), LAML (Tumor:6.79 ± 0.62, Normal: 3.66 ± 1.34, p = 5.3e−74), CHOL (Tumor:6.10 ± 0.94, Normal: 5.22 ± 0.25,p = 2.8e−3), we observed significant downregulation in seven tumors such as UCEC (Tumor: 6.45 ± 0.99, Normal: 7.04 ± 0.46, p = 5.7e−3), CESC(Tumor:6.56 ± 0.89. Normal: 7.33 ± 0.56, p = 1.2e−3), KIRP (Tumor: 6.03 ± 0.85, Normal: 6.43 ± 1.55, p = 1.8e−12), KIPAN (Tumor: 6.40 ± 0.94, Normal: 6.43 ± 1.55, p = 0.01), OV (Tumor: 6.44 ± 1.18, Normal: 7.15 ± 0.42, p = 1.2e−13), UCS (Tumor: 6.70 ± 0.93, Normal: 7.18 ± 0.39, p = 4.8e−3), KICH (Tumor: 5.68 ± 0.92, Normal: 6.43 ± 1.55, p = 1.8e−12). *SNHG15* is closely linked to marker genes for three classes of RNA modifications (m1A (10), m5C (13), and m6A (21)) genes, and the exact mechanism needs to be further explored, which confirms that *SNHG15* plays a major role in tumors (Fig. 8D).

### In vitro cell experiment results

The expression of *SNHG15* was measured by qRT-PCR in WT tissues and paired normal tissues (Fig. 8B). The siRNA interference of *SNHG15* significantly inhibited the expression of *SNHG15* compared with the control group (P < 0.05), as shown in Fig. 8C.

In addition, a series of experiments were performed to investigate the effects of *SNHG15* on the proliferation, invasion and migration of nephroblastoma cells. We investigated the effect of *SNHG15* on the proliferation and viability of nephroblastoma cells using CCK-8 assay (Fig. 8F). Wound healing assay showed that siRNA interference with *SNHG15* expression significantly inhibited G401 cell proliferation migration and promoted apoptosis compared with control (Fig. 8G). These results were further supported by Transwell assay, si*SNHG15* reduced the invasion and migration of nephroblastoma cells, as shown in Fig. 8E. In addition, we also detected the knock-down of *SNHG15* by Western blotting for the expression of EMT-related core proteins. Compared with the siNC group, knockdown of *SNHG15* decreased the expression of EMT-associated core protein (Fig. 8H). According to the results of cell cycle analysis, knocking down the expression of *SNHG15* resulted in its blockage in the S phase, which in turn inhibited the proliferation of tumor cells (Supplementary figure 1).

## Discussion

Nephroblastoma, also known as nephroblastoma (WT), is an embryonic tumor that is prevalent in children under 5 years of age. This disease accounts for 90% of renal tumors in pediatric patients. In recent years, the incidence of this disease has been increasing year by year in China, and it is a serious threat to the lives of children. Therefore, in this study, we performed a combined analysis of bulk RNA-seq and scRNA-seq to analyze the prognostic-associated lncRNAs in nephroblastoma and the heterogeneity among different cell types in nephroblastoma. *SNHG15* has been found to be an immune-associated prognostic biomarker in patients with nephroblastoma. Its high expression predicted poor prognosis, and high levels of M2 cell infiltration. *SNHG15* may promote M2 macrophage infiltration in nephroblastoma, which is potentially valuable in predicting prognosis and improving therapeutic efficacy, especially immunotherapy.

There are relatively few studies on lncRNAs in nephroblastoma. Most candidate lncRNAs are obtained from published articles, rather than the NGS technique. Besides, there is a lack of transcription profile identification and analysis of lncRNAs related to nephroblastoma. Teng et al. confirmed that lncRNA *MEG3* could significantly inhibit the proliferation, invasion, and migration of WT cells by regulating the Wnt/β-catenin signaling pathway [35]. Further, the expression of lncRNA *MEG3* was down-regulated in WT tissues and blood samples. Thus, it can be regarded as a potential tar-get for the diagnosis, treatment, and prognosis prediction of nephroblastoma. Wang et al. proved that the expression of lncRNA *SNHG6* increased in WT tissues and cells, and it could serve as the sponge of *miR-429* to up-regulate the expression of *FRS2*, promote the proliferation and glycolysis of WT cells, inhibit the apoptosis of WT cells, and accelerate the tumor progression of nephroblastoma[36]. Firstly, in this study, WGCNA analysis of prognosis-related lncRNAs was performed based on bulk RNA-Seq data of nephroblastoma in the TARGET database, and their functional enrichment was analyzed.

The data set from the TARGET database was adopted to identify the key modules related to the prognosis of nephroblastoma by WGCNA. Subsequently, the Kaplan–Meier survival analysis was performed on these genes in the modules based on the clinical data sets in the TARGET database. It can be validated that *SNHG15* correlated with the prognosis of nephroblastoma, and nephroblastoma patients with highly expressed *SNHG15* had a poor prognosis. In addition, the results of the ROC curve and univariate and multivariate Cox regression analysis suggested that highly ex-pressed *SNHG15* predicted a poor prognosis, and *SNHG15* can be regarded as an independent prognostic factor for the overall survival of these patients. In an attempt to further verify the function of *SNHG15*, an independent data set (GSE66405) was selected to verify the expression of *SNHG15* in nephroblastoma. The results confirmed that there were significant differences in the expression of *SNHG15* between WT tissues and normal kidney tissue. *SNHG15* can be identified as a key lncRNA in the pathogenesis of nephroblastoma.

*SNHG15* is a recently discovered lncRNA. There are fewer reports on the role and mechanism of *SNHG15* in the occurrence and development of tumors [37, 38]. At present, the exploration of *SNHG15* in nephroblastoma has not been reported at home and abroad. *SNHG15* is a snoRNA host gene with a length of 3,674 bp that can produce short-lived lncRNAs. It is located on human chromosome 7pl3, the upstream of myosin 1G gene, and contains five exon sequences. *SNHG15* is abnormally overexpressed in many tumors and can regulate gene expression and chromosome modification through the competing endogenous RNA (ceRNA) pattern or other mechanisms. Besides, it plays a vital role in the proliferation, migration, and invasion of tumor cells (39). Ma et al. demonstrated that knocking down *SNHG15* can inhibit the proliferation ability of pancreatic cancer cells in vitro and reduce the tumorigenicity in vivo [40]. Additionally, the RNA immunoprecipitation (RIP) assay results revealed that *SNHG15* inhibited the expression of *P15* and *KLF2* through *EZH2*-mediated *H3K27ME3*, and promoted the proliferation of pancreatic cancer cells. This indicated that *SNHG15* may be a potential biomarker for the early detection and individualized treatment. Chen et al. reported that knocking down the expression of *SNHG15* through siRNA can inhibit cell proliferation and invasion and induce apoptosis. Moreover, *SNHG15* can promote the proliferation and invasion of gastric cancer cells by regulating the expression of *MMP2* and *MMP9* proteins [41]. As per the results of this study, these nephroblastoma patients with highly expressed *SNHG15* had a lower overall survival rate and a higher recurrence risk. Meanwhile, the in vitro cell experiment results also suggested that down-regulating the expression of *SNHG15* can inhibit the proliferation and migration of tumor cells. These results indicated that *SNHG15* can be regarded as a biological marker to independently predict the prognosis of patients with nephroblastoma, which provided a new therapeutic target for the treatment of these patients.

The functional annotation and pathway enrichment analysis were also performed to further explore the biological processes related to *SNHG15*. The downstream target genes of *SNHG15* correlated with methyltransferase activity, DNA modifying enzyme, post-transcriptional regulation of gene expression, regulation of cellular amide metabolism, mRNA synthesis, and the processing, splicing and binding of RNA. Meanwhile, it was found that these downstream target genes were mainly involved in splice formation, *PI3K/AKT* signaling pathway, *IL-17* signaling pathway, *AMPK* signaling pathway, and cell cycle and apoptosis regulation. *AMPK* α subunits can be activated by the phosphorylation of the *AMPK* signaling pathway through liver kinase B1 (*LKB1*), Calcium/calmodulin-dependent protein kinase β (*CaMKK β*) and *TGF-β* activated kinase-1 (*TAK-1*). This would further inhibit energy-consuming biosynthetic pathway and activate the catabolic pathway related to ATP production, such as fatty acid oxidation and glycolysis. The substrates involved in apoptosis, protein synthesis, metabolism, and cell cycle can be phosphorylated by the *PI3K/AKT* signaling pathway to control key cellular processes. There is increasing evidence demonstrating that key epigenetic modifiers are directly or indirectly regulated by the *PI3K/AKT* signaling pathway, and they can participate in the *PI3K* cascade reaction in cancer. In recent studies, it has been revealed that there is a high level of phosphorylated AKT in the rat model of nephroblastoma [42, 43]. The proliferation and metastasis of nephroblastoma cells depend on the activation of the *PI3K/AKT* signaling pathway. Meanwhile, it has been found in this study that *SNHG15* negatively correlates with the expression of several key molecules in the *PI3K/AKT* signaling pathway [44]. These findings suggest that *SNHG15* may affect the occurrence and progression of nephroblastoma by activating the *PI3K/AKT* signaling pathway.

According to recent studies, the tumor immune microenvironment can affect the occurrence and progression of nephroblastoma. In this study, the correlation between *SNHG15* expression and the immune status of patients in the WT group was assessed by the ESTIMATE and ssGSEA algorithms. There were significant differences in the StromalScore and ESTIMATEScore between both groups. The immunological characteristics were assessed through the ssGSEA algorithm. The results showed that there was a dramatic difference in such immune cells as M2 macrophages, eosinophils, and neutrophils between both groups. According to some studies, the abnormal level of immune checkpoints may be an important contributing factor in cancer development. Therefore, the immune checkpoint genes were identified in this study, in an attempt to clarify whether there was a significant difference in these genes between both groups. Finally, the results showed that there were significant differences in *CD80*, *CD48*, *IDO2*, *CD276*, *CD28*, and *CD200R1* between both groups, which could be used as potential therapeutic targets for the treatment of nephroblastoma. These findings suggested that *SNHG15* may regulate the immune microenvironment of nephroblastoma, which would affect the progression of this disease and the effect of immunotherapy.

To verify the distribution and functional mechanisms of *SNHG15* in WT, we further analyzed the results at the single cell level and found that *SNHG15* was expressed in both tumor and immune cells. *SNHG15* could activate *PI3K/AKT* signaling and promote EMT or carcinogenesis in WT cells. Since M2 macrophage cell infiltration was higher in WT patients with high *SNHG15* expression, revealing its potential molecular mechanism on tumor cells; GSEA: "*HALLMARK-PI3K-AKT-MTOR -SIGNALING*", "Glutamatergic synapse" and "Glycolysis" pathways were highly enriched in the highly expressed *SNHG15* tumor cells. Highly enriched in *SNHG15* tumor cells. To study the cell–cell communication network between nephrogenic cell types under study, we applied CellChat (to scRNA-seq. Notably, Cancer cells and M2 macrophages interacted most with other cell clusters.

## Conclusions

In this study, scRNA-Seq and bulk RNA-Seq data were integrated to analyze the heterogeneity between different cell types in pediatric Wilms' tumor (WT) tissue. WGCNA and other bioinformatics approaches were used to explore high-throughput sequencing data and clinical data from nephroblastoma patients in the TARGET database and to validate them in in vitro experiments. Highly *SNHG15*-expressing cancer cells interacted with M2 macrophages at a higher frequency and intensity, and the key receptor-ligand pairs between them were mainly *NRXN3-NLGN1/NRXN1-NLGN1/NRG3-ERBB4/NRG1-ERBB4*.*SNHG15* may, through the *PI3K/AKT* signaling pathway promoting tumor invasion and metastasis, and it may be a new potential prognostic marker. This finding will be helpful for personalized treatment and clinical prognosis determination of nephroblastoma patients. This study still has some limitations. First, the lack of a pediatric nephroblastoma database, which is different from the adult nephroblastoma database that contains a large amount of transcriptomic and clinical data, makes it difficult to obtain more data for analysis. Secondly, due to different sequencing platforms and different microarray versions, the probe re-annotation approach could not completely cover all lncRNAs and inevitably missed some lncRNAs. Finally, more in vitro and in vivo experiments are needed to validate the specific role and mechanism of *SNHG15* in nephroblastoma.

### Supplementary Information

Below is the link to the electronic supplementary material

Supplementary file1 (TIF 1474 KB) Supplementary figure 1 Knocking down the expression of *SNHG15* resulted in its blockage in the S phase, which in turn inhibited the proliferation of tumor cells.

Supplementary file2 (DOCX 16 KB) Supplementary table 1 In this study, the primer sequences used for PCR.

## Data Availability

The datasets generated and analysed during the current study are available in the TARGET (Therapeutically Applicable Research to Generate Effective Treatments) database (https://ocg.cancer.gov/programs/target/data-matrix).
