# Supplementary figures and images for: Nephroblastoma-specific dysregulated gene SNHG15 with prognostic significance: scRNA-Seq with bulk RNA-Seq data and experimental validation
Source: Discov Oncol. 2024 Mar 25;15:87. doi: 10.1007/s12672-024-00946-w (PMC10963698; doi:10.1007/s12672-024-00946-w)

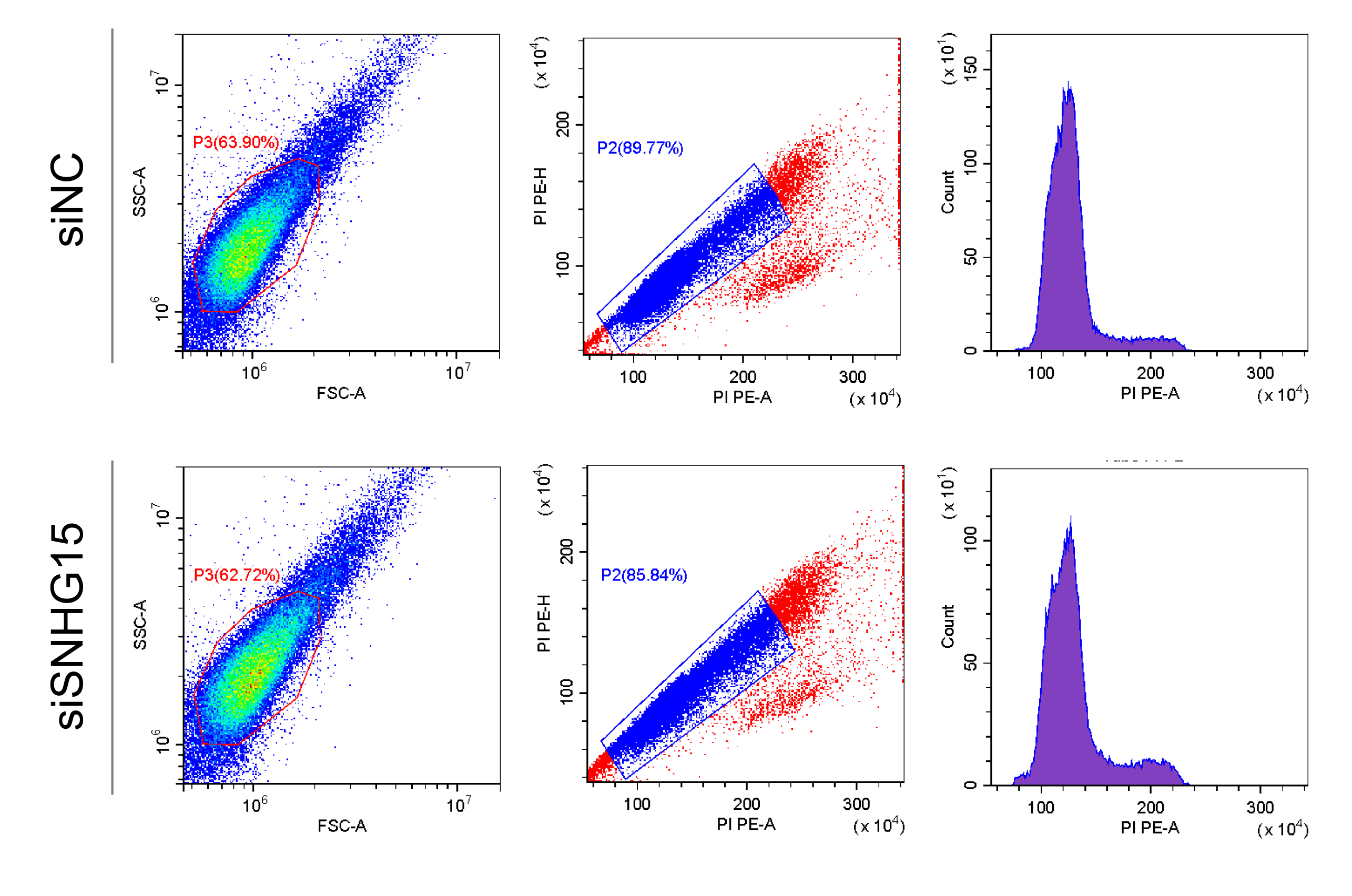

Supplement: Supplementary file 1 — Supplementary file1 (TIF 1474 KB) Supplementary figure 1 Knocking down the expression of SNHG15 resulted in its blockage in the S phase, which in turn inhibited the proliferation of tumor cells. [file 12672_2024_946_MOESM1_ESM.tif]
