# Supplementary material for: Nephroblastoma-specific dysregulated gene SNHG15 with prognostic significance: scRNA-Seq with bulk RNA-Seq data and experimental validation
Source: Discov Oncol. 2024 Mar 25;15:87. doi: 10.1007/s12672-024-00946-w (PMC10963698; doi:10.1007/s12672-024-00946-w)
Supplement: Supplementary file 2 — Supplementary file2 (DOCX 16 KB) Supplementary table 1 In this study, the primer sequences used for PCR. [file 12672_2024_946_MOESM2_ESM.docx]

Supplementary table 1: In this study, the primer sequences used for PCR.

| Gene symbol | F+R | Sequences |
| --- | --- | --- |
| SNHG15 | F | GTCTTCGGCAGTCTAGTCATC |
| SNHG15 | R | CTCTTCCACTTTGAGACCGTC |
| GAPDH | F | ATCACCATCTTCCAGGAGCGAG |
| GAPDH | R | GGGCAGAGATGATGACCCTTTTG |
